# Transplant Antennae and Host Brain Interact to Shape Odor Perceptual Space in Male Moths

**DOI:** 10.1371/journal.pone.0147906

**Published:** 2016-01-27

**Authors:** Seong-Gyu Lee, Kathy Poole, Charles E. Linn, Neil J. Vickers

**Affiliations:** 1 Dept. of Biology, University of Utah, Salt Lake City, UT 84112, United States of America; 2 Dept. of Entomology, Cornell University, Geneva, NY 14456, United States of America; INRA-UPMC, FRANCE

## Abstract

Behavioral responses to odors rely first upon their accurate detection by peripheral sensory organs followed by subsequent processing within the brain’s olfactory system and higher centers. These processes allow the animal to form a unified impression of the odor environment and recognize combinations of odorants as single entities. To investigate how interactions between peripheral and central olfactory pathways shape odor perception, we transplanted antennal imaginal discs between larval males of two species of moth *Heliothis virescens* and *Heliothis subflexa* that utilize distinct pheromone blends. During metamorphic development olfactory receptor neurons originating from transplanted discs formed connections with host brain neurons within olfactory glomeruli of the adult antennal lobe. The normal antennal receptor repertoire exhibited by males of each species reflects the differences in the pheromone blends that these species employ. Behavioral assays of adult transplant males revealed high response levels to two odor blends that were dissimilar from those that attract normal males of either species. Neurophysiological analyses of peripheral receptor neurons and central olfactory neurons revealed that these behavioral responses were a result of: 1. the specificity of *H*. *virescens* donor olfactory receptor neurons for odorants unique to the donor pheromone blend and, 2. central odor recognition by the *H*. *subflexa* host brain, which typically requires peripheral receptor input across 3 distinct odor channels in order to elicit behavioral responses.

## Introduction

The insect olfactory system is comprised of a peripheral structure that interacts with the fluid environment and captures odorous molecules that are then presented to receptor sites on olfactory receptor neurons [1]. The nature of the task of odor detection means that these interactions are specific. If every receptor were equally responsive to all odorants then no discrimination would be possible. In the pheromone systems of many moth species the peripheral receptors are highly specific, often for single pheromone components (i.e. single odorants) [2]. Since each receptor type communicates with a single olfactory glomerulus in the primary processing neuropil, the antennal lobe, the behaviorally active pheromone blend is represented by activity across more than one glomerulus [3]. The behavioral responses of moths often rely upon the presence of a blend of odorants and, as such, a function of higher olfactory pathways and processing in the brain must be to interpret the activity across olfactory glomeruli and construct a unified impression of the complete odor that interacted with receptors at the periphery.

For sympatric moth species, the ability to discriminate between con- and hetero-specific females is vital in maintaining reproductive integrity. Pheromone components released in the blend of one species (A) may contain the necessary constituents to attract males of a different species (B). However, additional components may be present in the pheromone of species A and when detected act antagonistically on the behavior of males of the other species (B). This is the case in Heliothine moths, a large group of cosmopolitan species spread widely around the globe [4,5]. Two species present in the United States, *Heliothis virescens* and *Heliothis subflexa* are closely related but females fail to attract heterospecific males because of differences in the pheromone requirements of males. Sex pheromonal attraction in male *H*. *virescens* necessitates activation of two separate olfactory pathways tuned to (*Z*)-11-hexadecenal (Z11-16:Ald) and (*Z*)-9-14-tetradecenal (Z9-14:Ald) respectively [6–11]. On the other hand, simultaneous activation of three separate olfactory pathways, tuned to Z11-16:Ald, (*Z*)-9-hexadecenal (Z9-16:Ald), and (*Z*)-11-hexadecenol (Z11-16:OH) respectively, is crucial to elicit attractive behavior in male *H*. *subflexa*, [12–14]. These differences are reflected in the odor sensitivity profiles of peripheral receptor neurons located on the antennae of each species [14].

**Fig 1:**
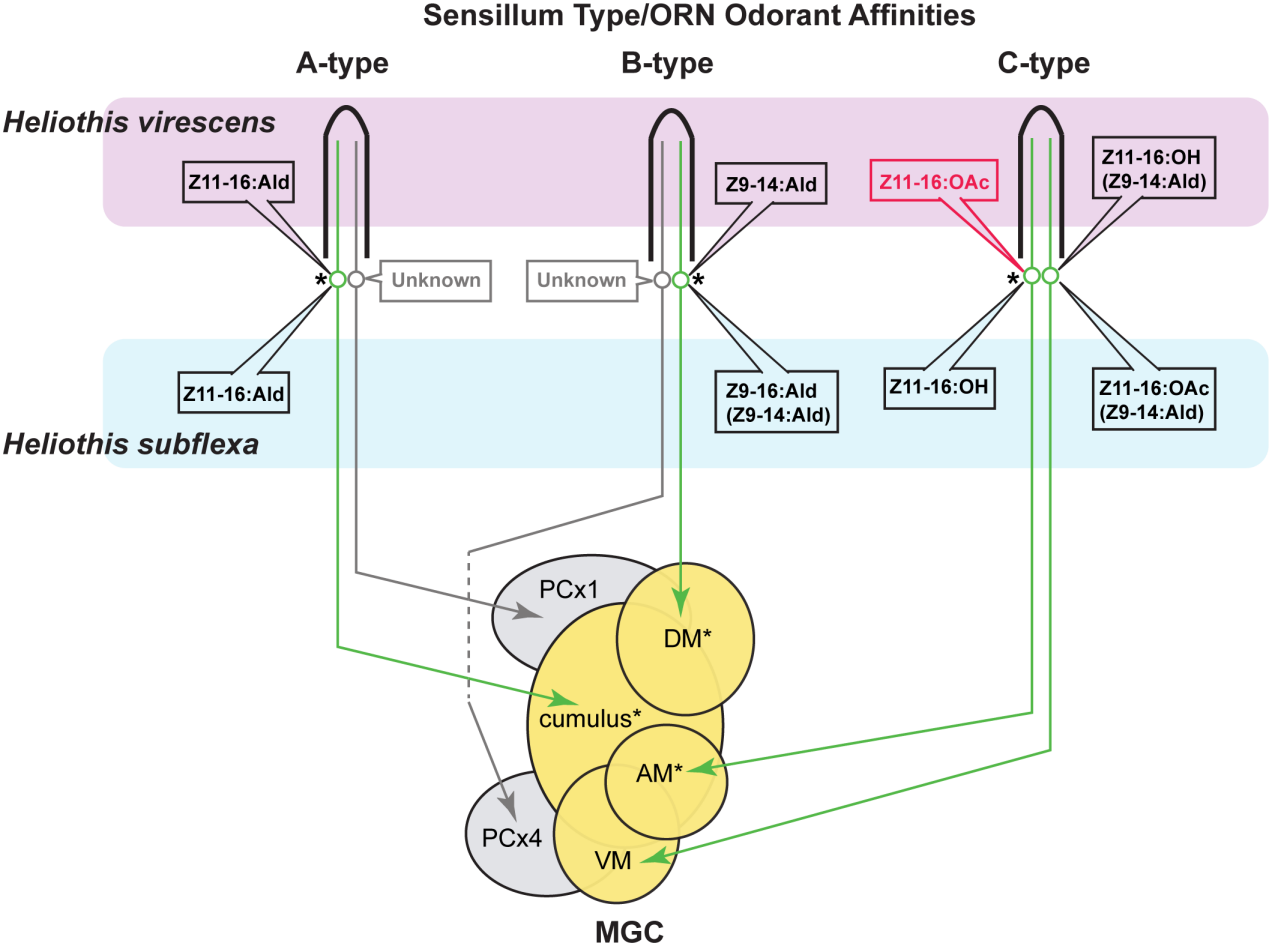
Organization of the peripheral olfactory system in adult *H*. *virescens* and *H*. *subflexa*. Pheromone sensitive olfactory receptor neurons (ORNs) are housed in three types of sensilla on the antennae of male *H*. *virescens* and *H*. *subflexa*. ORN axons project to antennal lobe glomeruli that constitute the macroglomerular complex (MGC) as well as part of a posterior glomerular complex (PCx). Behavioral preferences of males of the two species are accounted for by differences in the odorant affinites of olfactory receptor neurons in the B- and C-type sensilla in addition to higher order processing of these inputs. ORN affinities for odorants in brackets are weaker. An asterisk by individual ORNs indicates that the odorants associated with three pathways have behavioral significance. These pathways connect to three glomeruli in the MGC (cumulus, DM and AM, each also demarcated by an asterisk). The fourth MGC glomerulus (VM) is activated by odorants that have relatively little influence on behavior. The anatomy of the macroglomerular complex is indistinguishable between these two closely related species.

Within the antennal lobe, males of moth species belonging to different Lepidopteran families typically exhibit distinct numbers and arrangements of sexually dimorphic olfactory glomeruli that are involved in pheromone processing [15]. In the *Heliothis* and *Helicoverpa* genera, the spatial arrangements of glomeruli in which the axons of different pheromone specific receptor types terminate are similar [16,17] and indistinguishable in the two congeneric species used in this study, *H*. *virescens* and *H*. *subflexa* [7,13]. Thus, since these species utilize different pheromone blends, there are important differences between the actual odorant inputs to glomeruli in the same anatomical locations in the two species. Furthermore, these inputs have different “functionalities” attached to them, inasmuch as one glomerulus is associated with an odorant that antagonizes behavioral responses in one species while in the other it is associated with a different odorant that is essential to behavioral responses [7,13]. These features are summarized in Fig 1.

Transplantation of the antennal imaginal disc in moths has been utilized as a means of challenging the central olfactory neuropil with a novel set of olfactory inputs. Transgender antennal transplants to create gynandromorphic chimeras (animals with the antenna of the opposite sex) have been used to study behavioral, olfactory and developmental processes in the hawkmoth, *Manduca sexta* [18–21]. Vickers et al. [22,23] extended this technique by transplanting antennal imaginal discs across two related species (*H*. *virescens* and another Heliothine moth, *Helicoverpa zea*). The results of these various studies indicated that the donor antenna dictates the glomerular arrangement of the antennal lobe although some individuals with aglomerular or disorganized antennal lobe structure were still capable of odor-mediated flight [18,22–24]. Additionally, virtually all neurons (peripheral and central) appear to adopt the response profiles and glomerular associations of the donor antenna with some exceptions [23,25]. In the current study, we produced interspecies antennal imaginal disc transplants between *H*. *virescens* and *H*. *subflexa* to examine the interplay between changes in olfactory receptor input to the antennal lobe, central olfactory output to higher brain centers and resultant behavior. Our results indicate that it is a property of the host brain that dictates the number of required “channels” in order to recognize an odor “object” or “entity” as attractive. The behavioral requirements for different blends thus reflect not only the olfactory receptor repertoire characteristic of the normal donor antenna but also central constraints imposed by connections in the host brain.

## Results

### Behavior: *H*. *virescens* (donor antenna)–*H*. *subflexa* (recipient brain), V-S transplant males

**Fig 2:**
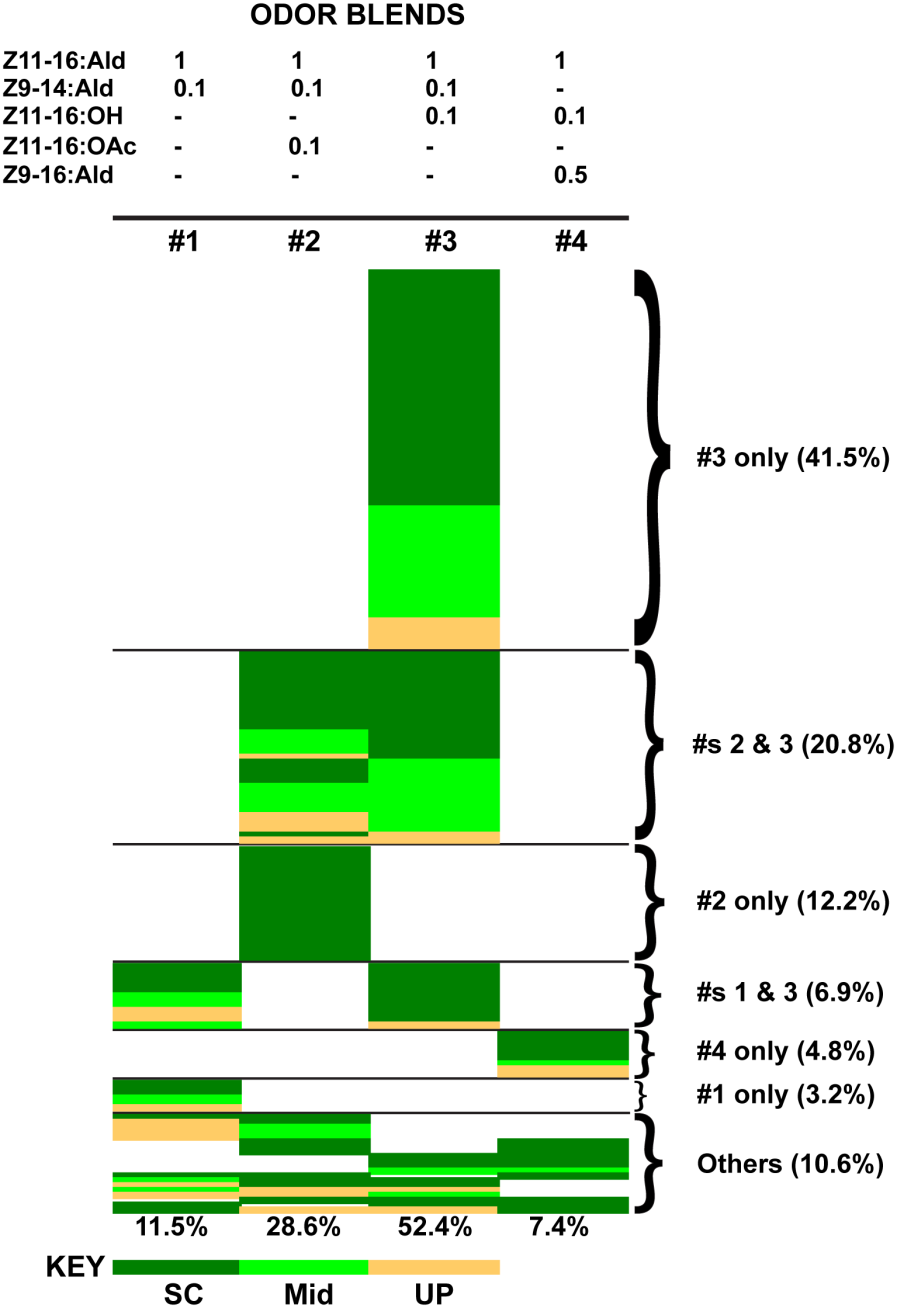
Behavioral responses of V-S (*H*. *virescens* donor antennal disc, *H*. *subflexa* host) transplant males. Transplant males exhibited a strong behavioral preference for blends containing Z11-16:Ald, Z9-14:Ald and either Z11-16:OH or Z11-16:OAc (blends 3 and 2 respectively). Over 70% of males that exhibited a behavioral response (N = 188) responded to one or both of these blends. Other males responded to either of the other two blends (#1, *H*. *virescens* blend; #4, *H*. *subflexa* blend) or to various combinations of the tested mixtures. Source contact (SC) is indicated by dark green, half way to the source (Mid) by a lighter green and upwind flight (UP) by orange. Numbers under each column represent percentage of total responses to that particular odor source (N = 269). Ratios of odorants in each blend are indicated above the four treatments.

A total of 365 *H*. *virescens* (donor antenna)–*H*. *subflexa* (recipient brain) transplant males (abbreviated V-S) were tested in the wind tunnel with all four different pheromone-related odor sources. Of the 365, 19 (5.2%) did not exhibit any movement from the release cage and were considered non-responders. From the group of 346 males that took flight from the release cage 158 (46%) neither oriented in the pheromone plume nor commenced upwind anemotactic flight, but rather flew in a random path at the downwind end of the wind tunnel. The remaining 188 transplant males (54%) exhibited the first in a sequence of orientation behaviors beginning with upwind oriented flight in the odor plume (UP), followed by flight to midway (MID) and then source contact (SC) in response to at least one of four odor sources (Fig 2). Out of the 188 pheromone source responders, 116 (62%) responded to only one of four odor sources, while the remaining 72 males (38%) showed responses to multiple sources. Six (3.2%) and nine (4.8%) V-S transplants responded only to the donor blend (*H*.*virescens* blend, treatment #1) and recipient blend (*H*. *subflexa* blend, treatment #4) respectively (Fig 2). Twenty-three of the 188 (12.2%) V-S transplant males were attracted only to treatment #2 (*H*. *virescens* blend + Z11-16:OAc), and 78 (41.5%) were attracted only to treatment #3 (*H*. *virescens* blend + Z11-16:OH). More than half of multiple source responders (39/72, 54.2%) were attracted to only two odor sources, treatment #s 2 and 3. Taken as a whole, the majority of responding V-S transplants (140/188, 74.5%) were attracted to either treatment #2 or 3 or both (Fig 2) and 81% of all responses were to these two treatments (N = 269 behavioral responses).

### Behavior: *H*. *virescens* (donor antenna)–*H*. *virescens* (recipient brain), V-V transplant males

**Fig 3:**
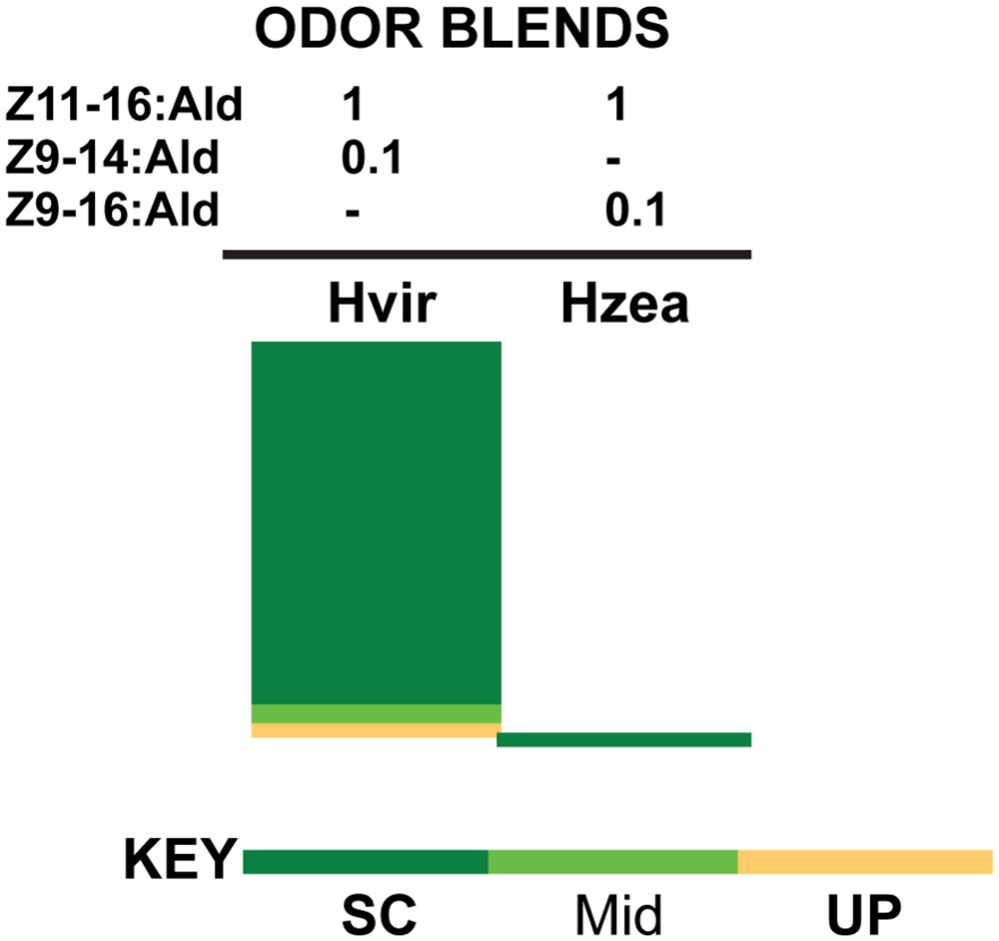
Behavioral responses of V-V transplant male controls. Of 43 males that exhibited positive behavioral responses, 42 responded only to the *H*. *virescens* blend (Z11-16:Ald + Z9-14:Ald, 1:0.1). Only a single male responded to the *H*. *zea* blend (Z11-16:Ald + Z9-16:Ald, 1:0.1). Source contact (SC) is indicated by dark green, half way to the source (Mid) by a lighter green and upwind flight (UP) by orange. Ratios of odorants in each blend are indicated above the four treatments.

A total of 131 control intra-species *H*. *virescens* to *H*. *virescens* (V-V) transplant males were tested in the wind tunnel to both control blends and initiated flight to at least one blend, indicating flight capability. Forty-three out of the 131 V-V males (32.8%) showed positive responses (upwind flight, flight to midway, or source contact) to one of the two odor sources. Only one male made source contact in response to the *H*. *zea* odor blend, but the remainder (42/43, 97.7%) was attracted to the *H*. *virescens* blend (Fig 3).

### Functional physiology and morphology of pheromone-responsive projection neurons: V-S transplant males

Among 367 V-S male transplants that were sent to Utah, intracellular recordings from olfactory PNs were attempted in 240 individuals. Successful recordings from a total of 37 PNs responsive to a single pheromone component were obtained in 32 individuals.

#### Z11-16:Ald PNs

**Fig 4:**
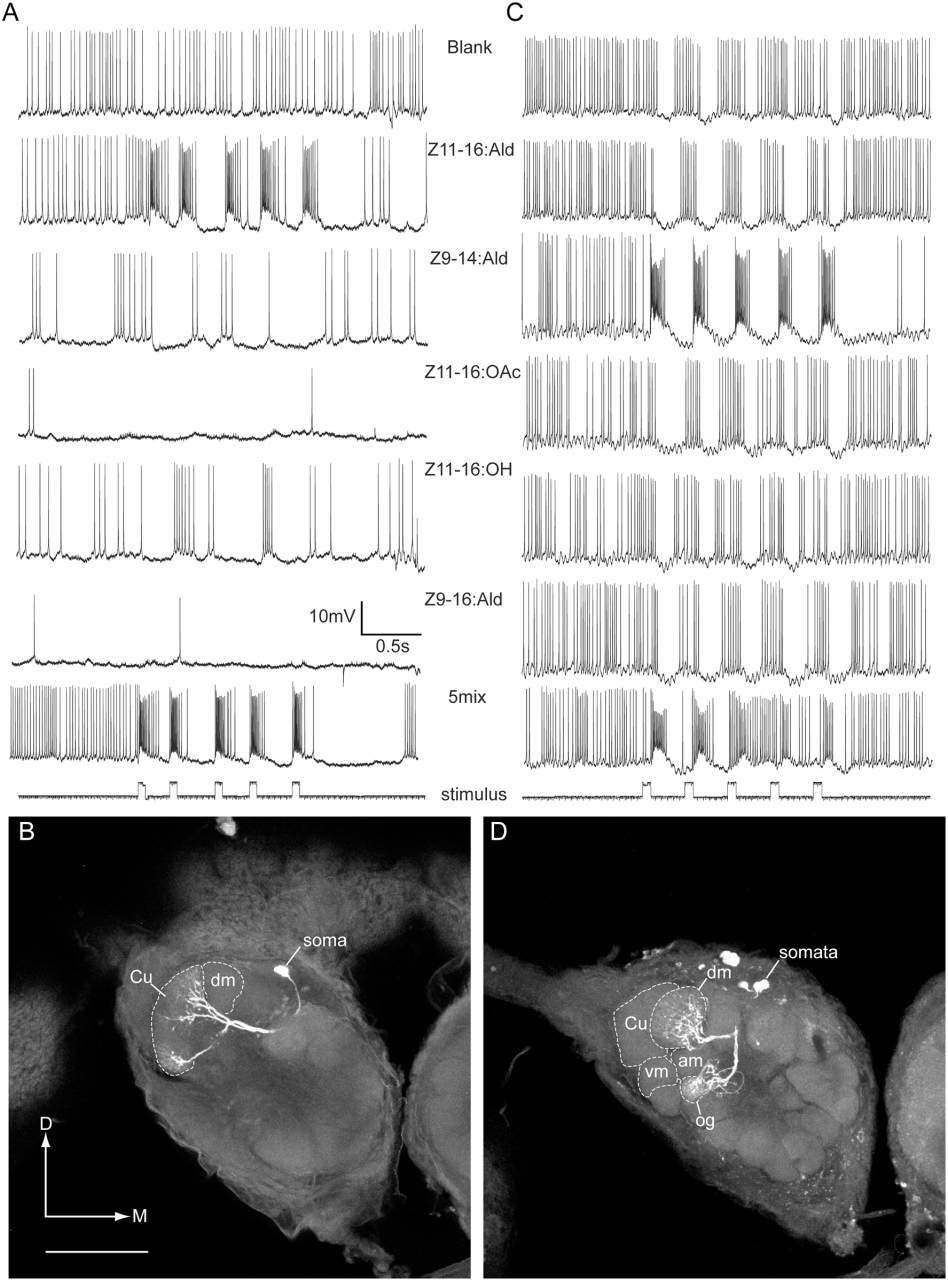
Physiology and morphology of transplant male projection neurons associated with ‘expected’ glomerular targets. Projection neurons (PNs) that responded specifically to stimulation with either Z11-16:Ald (A, B) or Z9-14:Ald (C, D). Both PNs had cell bodies located in the medial cell body cluster. Images (B, D) represent projections of a series of confocal images. Approximate locations of glomerular boundaries, which tend to be blurred in multiple image projections, are indicted by a dotted line. (A) Physiological profile of a neuron tuned to Z11-16:Ald. (B) A single neuron was stained in this preparation and shown to have a dendritic arborization restricted to the cumulus (Cu). The dorso-medial glomerulus (DM) is visible in this projection but the antero-medial and ventro-medial glomeruli (AM and VM respectively) have receded at this depth in the antennal lobe. (C) Physiological profile of a neuron tuned specifically to Z9-14:Ald. (D) Two PNs were stained in this preparation. One had a dendritic arbor restricted the DM glomerulus, consistent with Z9-14:Ald PNs in normal *H*. *virescens* males. A second neuron was localized to an ordinary glomerulus (og). In this more anterior image both AM and VM glomeruli are visible. Scale bar = 100μm (B, D); D, dorsal, M, medial.

**Fig 5:**
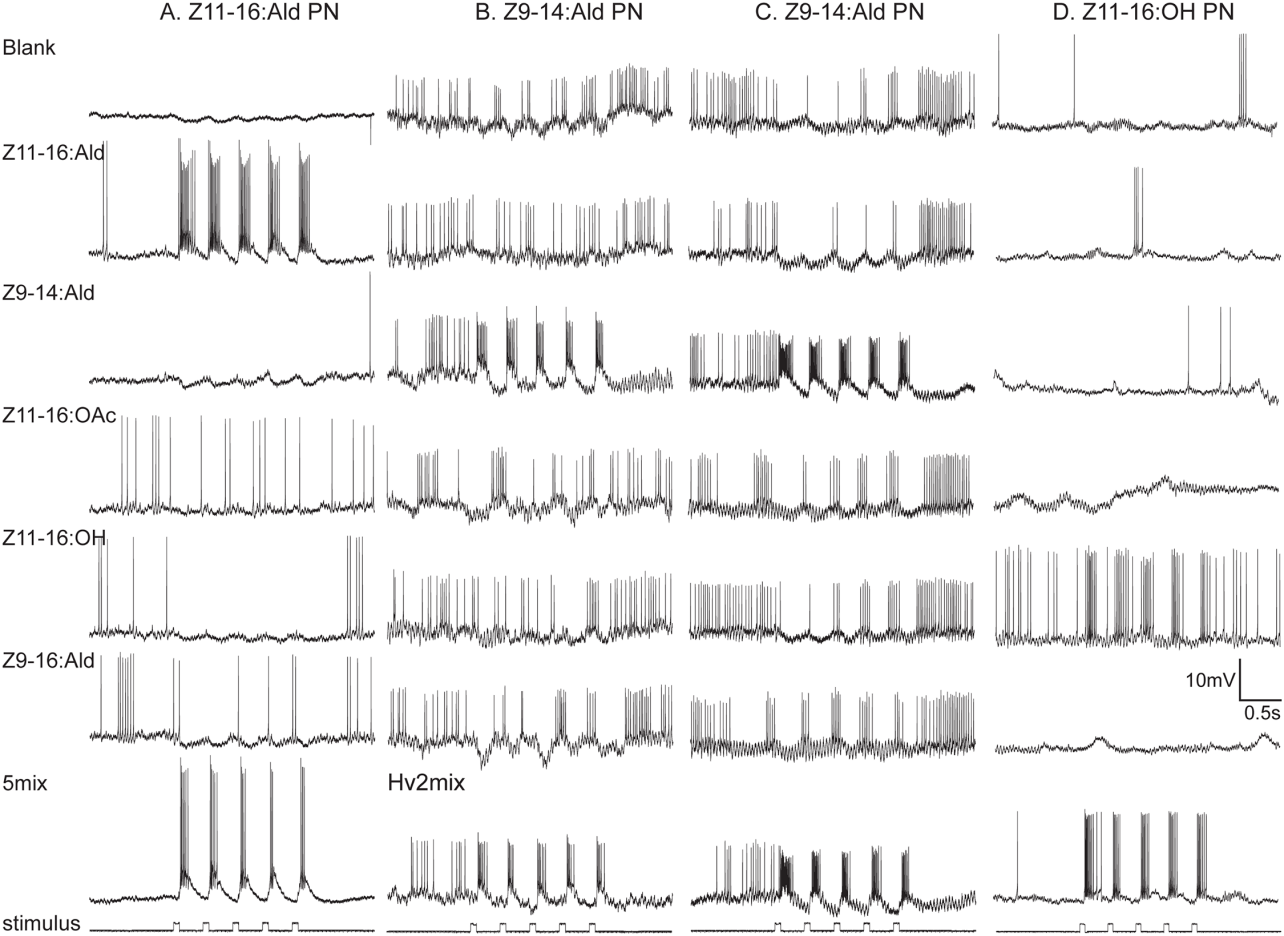
Physiology of the four projection neurons that accompany stained neurons in Fig 6. (A) Neuron with specific responses to Z11-16:Ald (morphology in Fig 6A). (B) From the same preparation, a PN that responded only to antennal stimulation with Z9-14:Ald (morphology in Fig 6B). (C) A PN that responded similarly to stimulation with Z9-14:Ald (morphology in Fig 6C). (D) PN responsive specifically to Z11-16:OH (morphology in Fig 6D).

**Fig 6:**
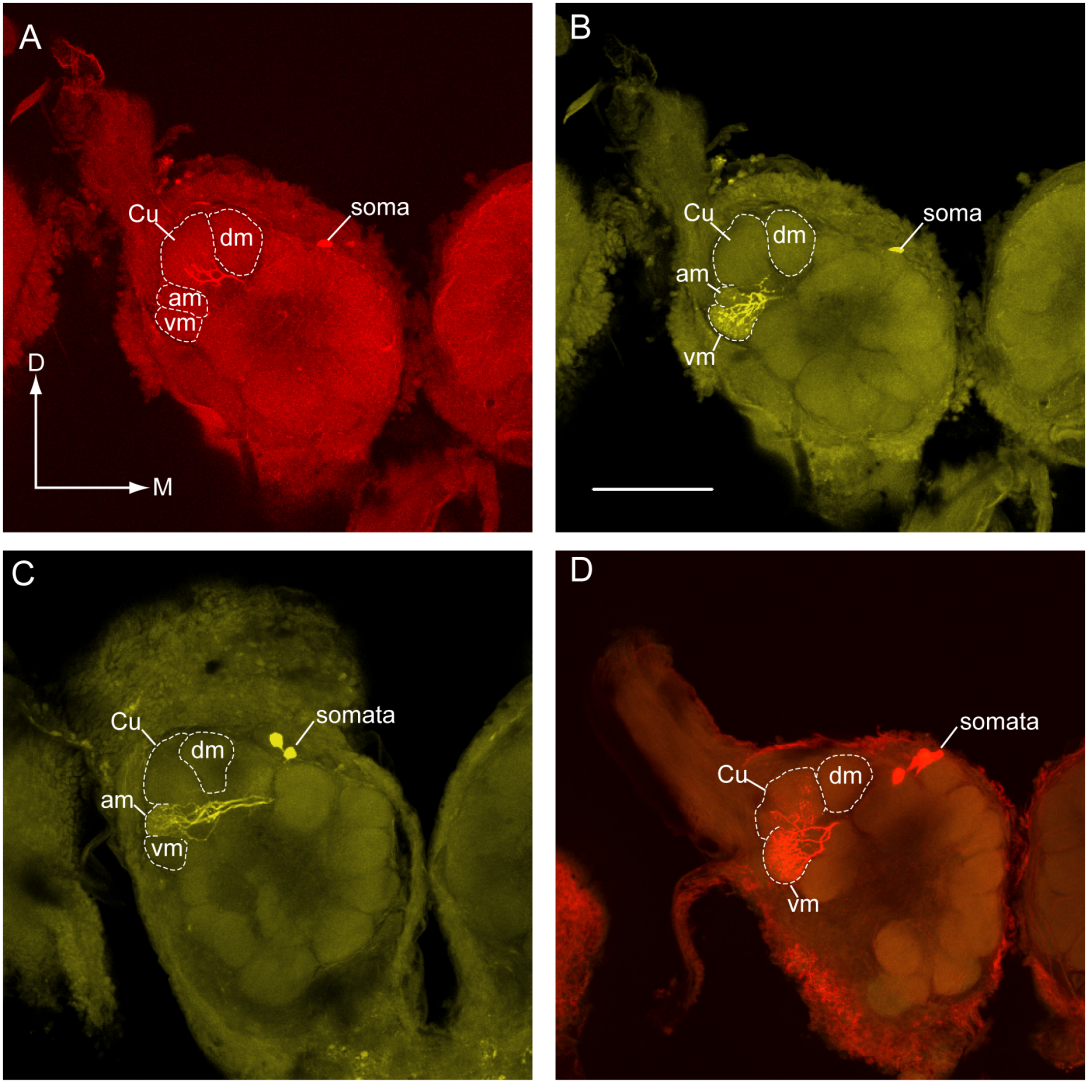
Projection neurons connected with the antero-medial (AM) and ventro-medial (VM) glomeruli had variable odorant associations in transplant males. (A) Z11-16:Ald projection neuron (PN) (stained with TMR-dextran) with a dendritic arbor restricted to the cumulus. (B) In the same preparation a Z9-14:Ald PN (stained with a different fluorescent marker–Lucifer yellow) exhibited a dense dendritic arbor restricted to the VM glomerulus. Note that some branches of this PN passage through the AM glomerulus but it was unclear if synaptic contacts were made within this glomerulus. (C) A Z9-14:Ald-specific PN associated with the AM glomerulus. In both B and C, dendritic arbors would be expected in the dorso-medial glomerulus (DM). (D) A Z11-16:OH PN exhibited a dense dendritic arbor in the VM glomerulus. Note that a second PN, associated with the cumulus, was also stained in this preparation. This PN responded to Z11-16:Ald. Scale bar = 100μm; D, dorsal, M, medial.

Recordings were made from 16 PNs that exhibited strong responses only to stimulation with Z11-16:Ald and blends that contained this odorant (Figs 4A and 5A). Stimulation with other odorants elicited phasic inhibition of spontaneous activity (Figs 4A and 5A), a response pattern associated with Type-2 Z11-16:Ald-responsive PNs [13]. In 6 preparations staining of these neurons was sufficient to visualize the glomerular location of their dendrites. The cumulus was the common dendritic target in all preparations (Table 1, Figs 4B and 6A). In four preparations multiple cell bodies in the medial cell body cluster were stained, and two of these preparations also exhibited staining in additional glomerular targets. Single-neuronal stainings were achieved from 2 preparations and both exhibited dendritic arbors restricted to the cumulus (Fig 4B).

**Table 1:** Summary of projection neuron recordings and successful staining in V-S (*H*. *virescens* donor antennal disc, *H*. *subflexa* host) transplant males. The number of preparations exhibiting a particular glomerular staining pattern is indicated.

Active Stimulant	Recordings	#	Glomerular Location of Stain				
			cumulus	DM	AM	VM	Others
Z11-16:Ald	16	2	+				
		1	+				
		1	+				
		1	+			+	+
		1	+			+	
Z9-14:Ald	16	2				+	
		1			+		
		2		+			+
		1					+
Z11-16:OH	4	2	+			+	

#### Z9-14:Ald PNs

In 16 preparations, PNs primarily responsive to Z9-14:Ald were physiologically characterized. These PNs exhibited strong bursts of action potentials followed by post-stimulus hyperpolarization in response to stimulation with Z9-14:Ald (Figs 4C, 5B and 5C). In six preparations PNs were successfully stained allowing visualization of their dendritic arbors and glomerular association. These PNs exhibited variable patterns of dendritic arborization. In two preparations arbors were visible in DM as well as one additional glomerulus–an ordinary glomerulus in one preparation and the PCx3 glomerulus in the other (Table 1, Fig 4D). DM is the normal glomerular target for Z9-14:Ald PNs in *H*. *virescens* males [7]. In another preparation, dendritic arbors were restricted to the AM glomerulus (Fig 6C). In two additional preparations, single PNs were stained in which the dendritic arborizations were restricted to the VM glomerulus (Table 1, Fig 6B). In the remaining preparation two cell bodies in the medial cell body cluster were stained with a single PN arborizing in a small ordinary glomerulus.

#### Z11-16:OH PNs

Recordings were made from 4 preparations in which neurons responded only to Z11-16:OH. These PNs exhibited phase-locked excitatory responses to stimulation with pulses of Z11-16:OH but no noticeable membrane hyperpolarization or depolarization was noted in response to other odorant stimuli (Fig 5D). PNs were successfully stained in two preparations. In both preparations, 5 medial cell body PNs were stained, and a strong dendritic arborization was observed in the VM glomerulus in both cases (Fig 6D). In each instance, recordings were also made from Z11-16:Ald PNs and a more weakly stained dendritic arborization in the cumulus likely reflected the staining of a second PN (Fig 6D).

### Functional physiology and morphology of pheromone-responsive projection neurons: V-V transplant males

Of the 103 V-V transplant males sent to Utah, intracellular recordings from olfactory PNs were attempted in 82 individuals. Thirteen interneurons were found to be responsive to one of the pheromonal odorants tested, and 7 preparations revealed successful neuronal staining sufficient to visualize the target olfactory glomeruli (Table 2).

**Table 2:** Summary of projection neuron recordings and successful staining in V-V (*H*. *virescens* donor antennal disc, *H*. *virescens* host) transplant males. The number of preparations that exhibited a particular pattern of glomerular association is indicated.

Active Stimulant	Recordings	#	Successful Stains			
			cumulus	DM	AM	VM
Z11-16:Ald	5	1	+			
		1	+			
		1	+			+
Z9-14:Ald	6	2		+		
Z11-16:OH	2	1				+
		1	+			+

#### Z11-16:Ald PNs

**Fig 7:**
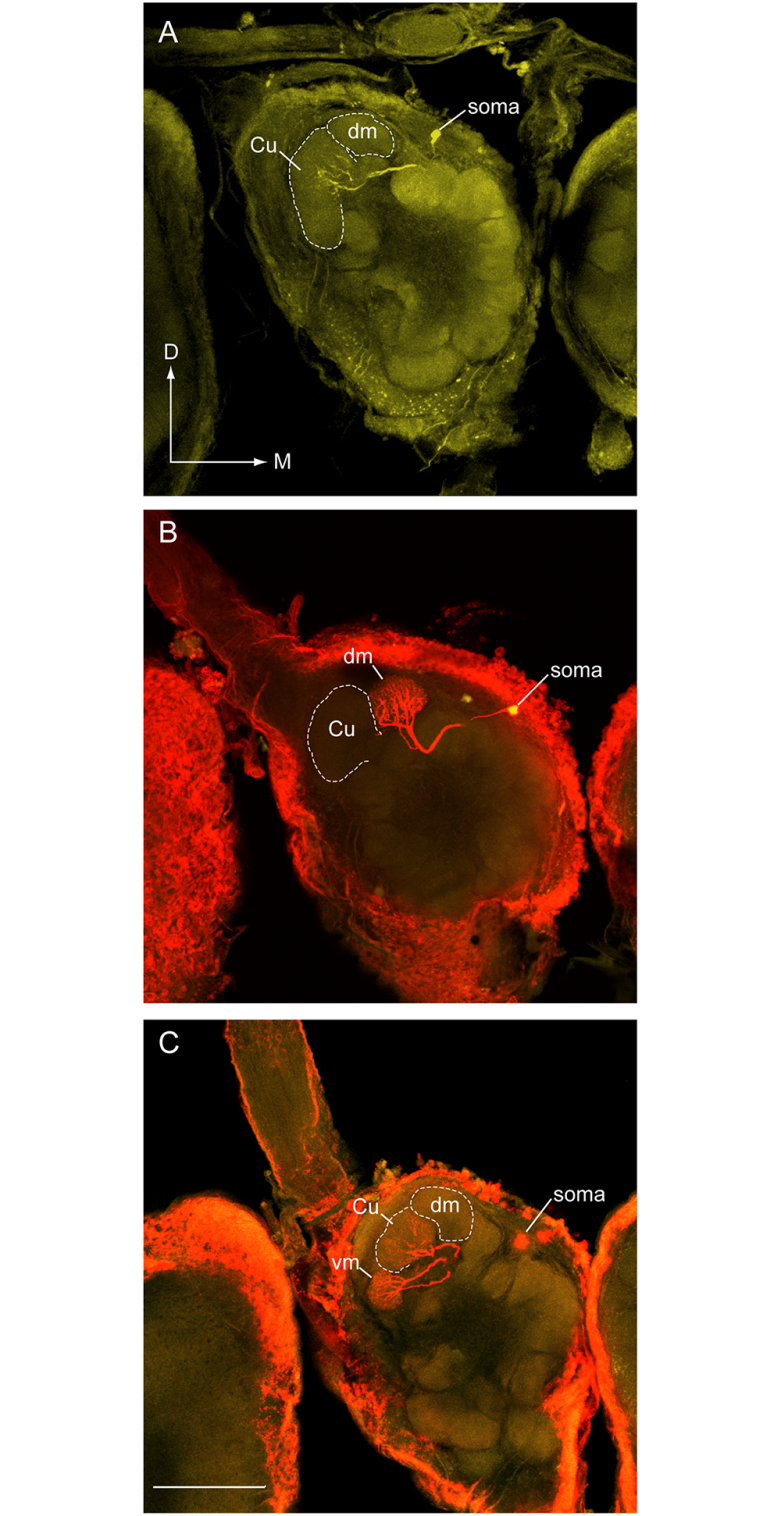
Projection neurons recorded from V-V control transplant males show a normal pattern of arborization in the MGC. (A) Medial cell body PN (MCB-PN) with an arbor restricted to the cumulus (Cu) responded to Z11-16:Ald. (B) MCB-PN with a dense dendritic arborization restricted to the dorso-medial glomerulus (DM) responded to stimulation with Z9-14:Ald. (C) In this preparation, two MCB-PNs with arbors restricted to the ventro-medial glomerulus (VM) and the cumulus were stained. The PN that responded to Z11-16:OH was associated with the VM glomerular location. Scale bar = 100μm; D, dorsal, M, medial.

**Fig 8:**
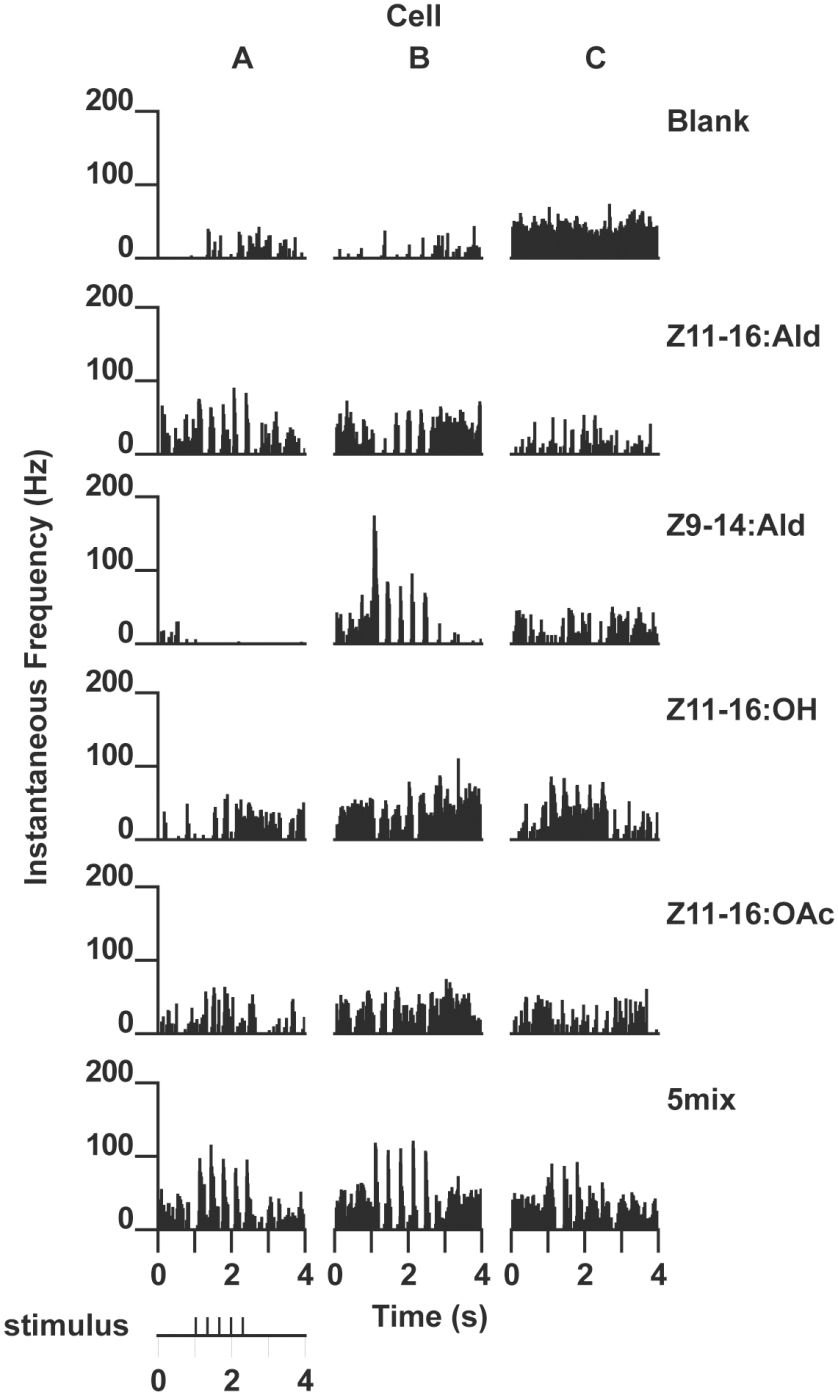
Physiological response profiles of the three projection neurons shown in Fig 7. Responses are shown as instantaneous frequency plots where responses to stimulation with 5 brief pulses of a specific odorant (or a mixture containing the odorant) result in volleys of action potentials seen as distinct peaks above background. (A) PN specifically responsive to stimulation with Z11-16:Ald or mixtures containing that odorant (morphology shown in Fig 7A). (B) PN specifically responsive to Z9-14:Ald or mixtures with this odorant (morphology shown in Fig 7B). Note strong hyperpolarizing responses to pulses of Z11-16:Ald and Z11-16:OH. (C) PN responsive to Z11-16:OH and blends that contained this odorant (morphology shown in Fig 7C).

Successful stains were achieved in 3 out of the 5 preparations in which attempts at labeling Z11-16:Ald-responsive PNs were made (Table 2, Fig 7A). These PNs exhibited excitatory bursts of action potential in response to stimulation with Z11-16:Ald and inhibitory responses to some of the other odorants, similar to the Z11-16:Ald Type-2 response pattern previously reported in normal *H*. *virescens* males [7] (Figs 7A and 8A). The cumulus was the common target glomerulus in all cases (Fig 7A), except for one preparation where dendritic arbors invaded the VM glomerulus in addition to the cumulus. Since two cell bodies were stained in this preparation, this staining pattern likely results from the glomerular associations of two different PNs.

#### Z9-14:Ald PNs

Six PNs with specific responses to Z9-14:Ald were encountered and in two preparations single PNs were successfully stained. Both had dendritic arborizations restricted to the DM glomerulus (Fig 7B). These PNs were strongly depolarized by presentation of Z9-14:Ald and inhibitory responses were observed to other odorants, particularly Z11-16:Ald (Fig 8B).

#### Z11-16:OH PNs

Recordings were made from Z11-16:OH-responsive PNs in two individuals. Stimulation with Z11-16:OH resulted in excitatory bursts of action potentials (Fig 8C). Spontaneous activity did not appear to be modulated by other odorants although stimulation with Z11-16:OAc resulted in membrane depolarization that was not sufficient to bring the neuron to threshold (Fig 8C). Neurons were successfully stained in both preparations. In one preparation 2 medial cell bodies were stained but only one dendritic arbor restricted to the VM glomerulus was visible. In the second preparation 2 medial cell bodies were also stained–in this case dendritic arborizations associated with these 2 PNs were visualized in the VM glomerulus and the cumulus (Fig 8C).

### Glomerular targets of pheromone-responsive ORNs: V-S transplant males

The single sensillum recording (SSR) and staining technique was used on 78 V-S transplants to verify the target glomeruli of primary olfactory receptor neurons (ORNs). In 59 individuals with at least one sensillum responding to one or more of a series of pheromone component stimulants, we encountered 81 physiologically active sensilla. Among them, 66 sensilla were categorized as belonging to the A-, B- or C-type sensilla types previously characterized for male *H*. *virescens* or *H*. *subflexa* [14]. Of these 25 were Z11-16:Ald-responsive A-type sensilla, 32 were identified as male *H*. *virescens* B-type sensilla, and 5 showed typical *H*. *virescens* C-type sensilla. Interestingly we also found 4 B-type sensilla of male *H*. *subflexa* from three V-S transplants. However, the response profiles of the remaining 15 of 81 physiologically active sensilla did not fit either the A-, B-, or C sensillum types found in previous studies. A total of 59 sensilla were treated with cobalt dye to stain physiologically characterized ORNs. Of these, 44 sensilla showed response patterns typical of pheromone-responsive sensilla recorded previously in male *H*. *virescens*.

#### A-type sensilla

**Fig 9:**
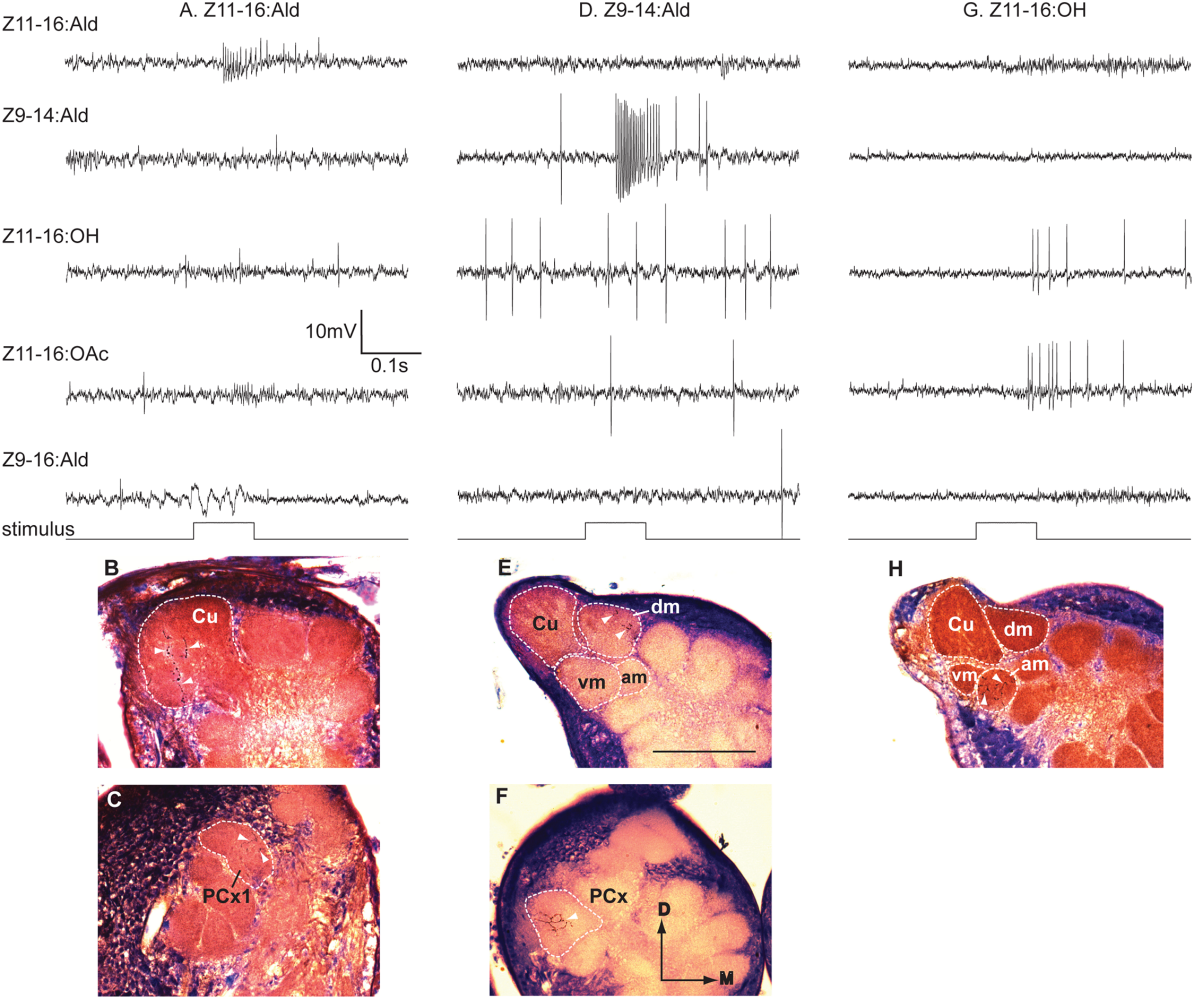
Response profiles and glomerular targets of olfactory receptor neurons (ORNs) from long trichoid sensilla on the antennae of transplant males. Recordings from V-S transplant males confirmed the presence of sensillar types that are typical for a normal *H*. *virescens* antenna. (A-C) Recording from a A-type sensillum housing a Z11-16:Ald ORN (A). Staining revealed projections to the cumulus and PCx1 glomeruli (B, C). (D-F) Recording from a B-type sensillum containing a Z9-14:Ald ORN (D). Staining revealed projections from this sensillum to the dorso-medial glomerulus (DM) and a posterior complex glomerulus (PCx) (E, F). (G-H) Recording from a C-type sensillum containing ORNs responsive to Z11-16:OAc and Z11-16:OH (G). Staining revealed a projection to the antero-medial glomerulus (AM) in this preparation–an observation that is consistent with the normal arborization pattern of ORNs associated with this sensillum type (H). The other paired ORN in the C-type sensillum typically projects to the ventro-medial glomerulus (VM) (but stains of axonal terminals in VM were not seen clearly in this preparation). Some silver-intensified cobalt stains of axon terminals are indicated by small white arrowheads in each section. Scale bar = 100μm (for histological images: B, C, E, F, H); D, dorsal, M, medial–axes the same for each histological image.

Normal A-type sensilla in V-S transplant males housed an ORN that responded exclusively to odor stimulation with Z11-16:Ald by generating a volley of spikes above background firing rates and responses observed to control pulse stimulation (Fig 9A). Six out of 17 staining attempts for A-type sensilla allowed identification of axonal targets in the antennal lobe. In five preparations staining revealed either one ORN with axonal arbors restricted to the cumulus or two ORNs with axons terminating in the cumulus and PCx1 (Table 3, Fig 9B and 9C) as is typical for normal *H*. *virescens* males. The odorant ligands for ORNs that terminate in the posterior complex are currently unknown [26,27]. Staining of one A-type sensillum gave rise to axon terminals in the cumulus, DM, and PCx4.

**Table 3:** Summary of recordings from each sensillum type (A, B, and C) on V-S (*H*. *virescens* donor antennal disc, *H*. *subflexa* host) transplant males. Total recordings for each sensillum type are indicated as well as the number of staining attempts. The specific number of successful olfactory receptor neuron stains and their associations with particular glomeruli are indicated.

Sensillum Type	Staining attempts/ Recordings	#	Glomerular Location of Stain					
			cumulus	PCx1	DM	PCx4	AM	VM
A-type	17/25	3	+	+				
		2	+					
		1	+		+	+		
		1		+				
B-type	23/32	2			+	+		
		2			+			
		1	+	+	+	+	+	+
C-type	4/5	1					+	+
		1					+	

#### B-type sensilla

Like most normal B-type sensilla found in male *H*. *subflexa* [14,27] and *H*. *virescens* [14] V-S transplant B-type sensilla could be identified by the unique wave forms of two ORNs housed therein. The wave forms of Z9-14:Ald-responsive ORNs are more symmetric, but non-responsive action potentials from a secondary ORN display a larger hyperpolarizing potential. A rapid burst of action potentials in response to stimulation with Z9-14:Ald was noted in these recordings (Fig 9D) with no responses to other stimuli. This physiological profile is consistent with that of B-type sensilla in *H*. *virescens* males (in *H*. *subflexa* males, one ORN responds to both Z9-14:Ald and Z9-16:Ald). Staining of five B-type sensilla (23 attempts) were successful, with four preparations exhibiting typical B-type sensillum ORN pathways to DM and PCx4 (Table 3, Fig 9E and F), and one exhibiting a staining pattern characteristic of A-, B-, and C-type sensilla simultaneously (cumulus, PCx1, DM, PCx4, AM, and VM).

#### C-type sensilla

We found 5 typical male *H*. *virescens* C-type sensilla, with two ORNs responsive to Z11-16:OAc and Z11-16:OH (weak responses to Z9-14:Ald) respectively [11,14] (Fig 9G). In 2 out of the 4 attempts ORNs were successfully stained. In one case both the VM and AM glomeruli were targeted (Fig 9H) whereas in the other axonal arbors were visible in the AM glomerulus only.

#### Atypical pheromone-responsive sensilla

Of the 15 sensilla that showed atypical response patterns, 6 ORNs were strongly excited by Z9-14:Ald and weakly by the other pheromone components. One of two sensilla that responded strongly to Z9-14:Ald (weak responses to the other 3 odorants; Z11-16:OH, Z11-16:OAc, and Z9-16:Ald) exhibited 4 stained ORNs arborizing in the cumulus, PCx1, DM, and AM. We found 6 atypical types of sensilla with responses evoked only by Z11-16:OAc. Of 5 cobalt staining attempts with these sensilla, one preparation labeled PCx2. We also found three long trichoid sensilla that exhibited specific responses only to Z9-16:Ald from two V-S transplants, which had not been observed previously in normal *H*. *virescens* males. One of two preparations with cobalt dye application appeared to stain one ORN targeting PCx4.

## Discussion

In this paper we present results from transplantation experiments in which the pre-imaginal antenna of one moth species was transplanted and then developed connections with the primary olfactory processing center in the brain of a different species during metamorphosis. Normal male adults of the two species between which transplant experiments were conducted differ fundamentally in their odor mixture composition requirements for conspecific female recognition. *H*. *virescens* and *H*.*subflexa* males require pheromone blends comprised of 2 and 3 components respectively. These blends differ in their constitutive odorants sharing only the most abundant (major) pheromone component, Z11-16:Ald. The majority of *H*. *subflexa* males with a transplanted *H*. *virescens* antenna (V-S transplants) only responded to one of two 3-component blends that were not attractive to normal males of both species: Z11-16:Ald, Z9-14:Ald and either Z11-16:OH (41.5%, Fig 2) or Z11-16:OAc (12.2%, Fig 2). Our results show that both three-odorant combinations stimulated three distinct receptor pathways in these transplant males, and imply that the host *H*. *subflexa* brain retained its intrinsic requirement for the activation of three separate odorant channels in order to form the percept of a “conspecific” odor blend and elicit behavioral responses. In transplant males connections were made between peripheral ORNs and central projection neurons in olfactory glomeruli arranged in a macroglomerular complex with a spatial arrangement that appeared identical to that usually seen in normal adult males of the two species. Stained ORNs in transplant males projected to glomeruli that adhered to a donor-based map of odorant affiliations. However, while many recipient PNs were associated with the expected glomerular locations, others targeted glomerular locations different than those expected based on their odorant response profile indicating flexibility in the functional characteristics of the spatial map. Thus, central pathways exhibited some plasticity in assembling both predicted as well as novel odorant combinations into cognate groupings sufficient to stimulate behavioral responses.

### Channel 1: Z11-16:Ald–Cumulus

Previous reports on the olfactory pathways of sex pheromone responsive neurons in both species concluded that the cumulus was the primary processing center in the AL for Z11-16:Ald information [6,7,10,11]. Of the six successfully labeled A-type sensilla in V-S transplants all targeted their axons to the cumulus. Intracellular staining of Z11-16:Ald-responsive PNs in both V-S transplants and control V-V transplants (Figs 4 and 7) also revealed consistent labeling within the cumulus. With the exception of two species, *Helicoverpa assulta* and *Helicoverpa gelotopoeon* [28–30], Z11-16:Ald is utilized as the major pheromone component in all other heliothine species. In the most intensively studied three North American Heliothinae, *H*. *zea*, *H*. *virescens*, and *H*. *subflexa* Z11-16:Ald ORNs are co-localized with PCx1-targeting ORNs in the A-type sensilla and project to the cumulus of the MGC [26,27]. This pattern appeared to be unaffected by either intra- or inter-specific transplant procedure.

### Channel 2: Z9-14:Ald

One noticeable characteristic evident from the behavioral responses of V-S transplant males toward two pheromone blend mixtures (#2 and #3) revealed that Z9-14:Ald had displaced Z9-16:Ald as an essential secondary component. The DM glomerulus is the target of B-type sensilla ORNs for the secondary pheromone components, Z9-14:Ald and Z9-16:Ald in *H*. *virescens* and *H*. *subflexa* respectively [10,27]. Hybrid males created by crossing *H*. *subflexa* with *H*. *virescens* are unable to discriminate behaviorally between Z9-14:Ald and Z9-16:Ald because the DM-targeting B-type ORNs are responsive to both odorants [31–34]. Thus, the molecular mechanisms that underlie B-type ORN–central PN targeting to the DM glomerulus are likely conserved across these two species and our results from V-S transplant males indicated that Z9-14:Ald ORNs targeted the DM glomerulus. Therefore, we predicted that the central neuronal pathways usually associated with the DM glomerulus (normally responsive to Z9-16:Ald ORN input) would now be activated by Z9-14:Ald-responsive ORNs originating on the donor antenna. However, even though inclusion of Z9-14:Ald was necessary to drive behavioral responses, stains of the corresponding Z9-14:Ald PNs in V-S transplant males revealed a more liberal pattern of glomerular association than is typical for these systems [6,7]. Uniglomerular arborizations were associated not only with the expected DM glomerulus but also the AM and VM glomeruli in different V-S preparations. Similarly, Vickers et al. [23] noted inconsistencies in the glomerular-associations of Z9-14:Ald PNs (normal donor physiology) in transplants between *H*. *virescens* and another species, *Helicoverpa zea* (V-Z transplants).

In *Drosophila melanogaster*, PNs have been shown to play a primary role in establishing the antennal lobe locus into which their cognate partner ORNs subsequently grow during metamorphosis [35,36]. Overexpression of Dscam in specific groups of PNs resulted in spatial shifts in the location of glomeruli but connections were still formed with appropriate partner ORNs [35]. Hence, shifts in spatial location of particular odorant-glomerular affiliations do not necessarily change the functional value of a particular odorant. Apparent mismatches in ORN input–PN output have been reported in the moth, *Trichoplusia ni* [37], but this study only examined the location of PN dendrites and did not account for the possibility that cognate ORNs may have also shifted their spatial glomerular targets. In the European corn borer, *Ostrinia nubilalis* the glomerular sites for processing two pheromone components in hybrid E- and Z-strain males as well as backcross males was not directly correlated with behavioral preferences [38,39]. This study again demonstrated that flexibility existed along the axis of spatial location–functional value for a particular odorant.

### Channel 3: Z11-16:OH and Z11-16:OAc

The significance of a third channel in mediating transplant male behavioral responses was highlighted by the small number of responses to a typical *H*. *virescens* 2-component blend (Z11-16:Ald plus Z9-14:Ald) in comparison to the large group of males that responded to 3-component blends (Z11-16:Ald, Z9-14:Ald plus Z11-16:OH/Z11-16:OAc).

Consistent with the pattern in normal *H*. *subflexa* males a preponderance of V-S transplant males responded only to a single 3-component blend containing Z11-16:OH (41.5%). A second, smaller group of males also responded to only one 3-component blend in which Z11-16:OH was omitted and Z11-16:OAc included (12.2%). Furthermore, a subset of males responded to both 3-component blends (20.8%). Since *H*. *subflexa* males showed a complete lack of response to a 3-component mixture comprised of Z11-16:Ald, Z9-16:Ald and Z11-16:OAc [12] responses of transplant males to this blend indicated that Z11-16:OAc was able to substitute for Z11-16:OH in activating a third central channel, a departure from the usual odor preferences exhibited by *H*. *subflexa* males.

Although normal males of both species detect Z11-16:OAc (through the C-type sensillum, Fig 1), transplant male responses to the 3-component blend containing this odorant were not anticipated because it is behaviorally either antagonistic (*H*. *virescens*) or benign (*H*. *subflexa*). In both *H*. *subflexa* and *H*.*virescens* males C-type sensilla project one axon to the AM glomerulus and one to the VM glomerulus. In *H*. *subflexa* Z11-16:OH PNs were associated with the AM glomerulus and, by exclusion, Z11-16:OAc PNs with the VM glomerulus [13] with the opposite configuration in *H*. *virescens* (Fig 1). Thus, both species have 3 behaviorally relevant pathways associated with the cumulus, DM and AM glomeruli as well as a 4^th^ receptor pathway associated with the VM glomerulus that is relatively benign with respect to behavior (Fig 1). While all four receptor input channels appeared able to participate in mediating attraction of Z11-16:OAc-responding transplant males, the behavioral responses of these males were consistent with the requirement for activity across 3 central channels. Such a result could be due to convergence of two receptor pathways in the same glomerulus similar to that seen in normal males of several *Helicoverpa* species [17,26]. This seems unlikely in transplant males since the 4-compartment MGC glomerular arrangement was not compromised and ORN stains of C-type sensilla revealed terminals in the AM and VM glomeruli. Alternatively, PNs with multiglomerular dendritic inputs could allow responses to Z11-16:OH and Z11-16:OAc along a spectrum that modulates behavioral responses to both blends. In the current study two Z11-16:OH-responsive PNs were identified in separate V-S transplant males and both targeted only the VM glomerulus (Fig 5, Table 1). Such a pattern coincides with the configuration normally seen in *H*. *virescens* males (NV: unpublished obs.). No PNs were identified with either unusual response profiles or multiglomerular arborization patterns. The report of a single PN with a novel response profile displaying an unexpected multiglomerular dendritic arborization in donor-induced glomeruli (AM & VM) in a previous study of V-Z transplants [23] lends some credence to this explanation.

Moth pheromone detection is a labeled line system in that individual glomeruli are dedicated to receive input from highly specific sensory neurons typically tuned to one or two specific monomolecular odorants. But activity across the glomerular array is necessary because pheromone blends and the blends released by heterospecific females are mixtures. Thus, the attractiveness of a pheromone blend (or potential aversiveness of a heterospecific blend) is usually dependent upon integration of activity across different labeled lines and, hence, glomeruli. Our results demonstrate that the precise spatial pattern of glomerular activation can be somewhat flexible provided that the correct recognition or percept “essential” channels are connected and appropriately activated by sensory input. Our understanding of the molecular and developmental mechanisms that underpin appropriate connections between olfactory ORNs and PNs continues to grow (e.g. [36,40]). Further investigations into the formation of specific connections made between neurons both within the antennal lobe as well as higher brain centers will continue to yield important insights into odor perception.

## Materials and Methods

### Insects

Colonies of *H*. *virescens* and *H*. *subflexa* were maintained at the Geneva, N.Y. laboratory following procedures outlined by Jurenka et al. [41] (16:8 L:D photoperiod, 25°C, 40–50% RH).

### Transplantation Procedure

The transplant procedure was initially developed by Schneiderman et al. [18] for gynandromorphic transplants (male–female) in the large sphingid moth, *M*. *sexta* and modified by Linn et al. [42] for the much smaller crambid moth, *Ostrinia nubilalis*. Vickers et al. [22,23] performed antennal imaginal disc transplants between a pair of Heliothine moths: *H*. *zea* and *H*. *virescens*. In the experiments reported here, antennal imaginal discs were transplanted between male *H*. *virescens* and *H*. *subflexa* larvae (V-S transplants). In addition, control transplants between male *H*. *virescens* were also performed (V-V transplants). The sex of individual larvae was ascertained [43] and imaginal discs were transplanted in the middle of the final larval instar. Larvae were restrained in aluminum tubes and anesthetized in ice water. The antennal imaginal disc is located below the cuticular surface of the larval antenna. Both antenna and disc were removed and placed in a drop of chilled saline solution. The transplant recipient was prepared by removing the right antenna and disc before the donor disc was inserted and sealed with VetBond^™^(3M). Following the operation each recipient was placed in an individual 50 ml plastic cup containing fresh media. Recipient larvae were inspected on a daily basis for activity, feeding, and subsequent pupation. Between 2–3 days post eclosion of the adult moth and one day before behavioral testing, the left (normal) antenna was excised at the base.

### Chemicals (behavior)

Synthetic pheromone-related compounds (*Z*)-11-hexadecenal (Z11-16:Ald), (*Z*)-9-hexadecenal (Z9-16:Ald), (*Z*)-9-tetradecenal (Z9-14:Ald), (*Z*)-11-hexadecenyl acetate (Z11-16:OAc), and (*Z*)-11-hexadecenol (Z11-16:OH) were obtained from either Bedoukian Research, Inc., Danbury, CT, or Pherobank (purities > 95%; verified by gas chromatography) and prepared as stock solutions in HPLC grade hexane. Blends were created by admixing individual components on 5 x 9 mm rubber septa (A.H. Thomas Co.). Septa were soaked in hexane for 24 h prior to use. The following blends were utilized for testing V-S transplant males with Z11-16:Ald loaded at 200μg and additional components added according to the stated ratio:

1. Z11-16:Ald + Z9-14:Ald (1:0.1) (*H*. *virescens* blend)
2. Z11-16:Ald + Z9-14:Ald + Z11-16:OAc (1:0.1:0.1)
3. Z11-16:Ald + Z9-14:Ald + Z11-16:OH (1:0.1:0.1)
4. Z11-16:Ald + Z9-16:Ald + Z11-16:OH (1:0.5:0.1) (*H*. *subflexa* blend).

Treatments 2 and 3 represent the *H*. *virescens* blend augmented by an additional component. In each case, the 3-odorant mixture would not be expected to be attractive to normal *H*. *virescens* males because the presence of the additional odorant is aversive to males. These blends would also be unattractive to *H*. *subflexa* males because they lack the requisite components, Z9-16:Ald (treatments 2 and 3) and Z11-16:OH (treatment 2).

Testing of control (V-V) males was conducted using the following blends:

1. Z11-16:Ald + Z9-14:Ald (1:0.1) (*H*. *virescens* blend)
2. Z11-16:Ald + Z9-16:Ald (1:0.1) (*H*. *zea* blend)

Treatment 2 represents the blend of another Heliothine moth (*Helicoverpa zea*) and was used to determine if the transplant procedure introduced behavioral responses that might reflect novel receptor capabilities at the antennal level [25].

### Flight Tunnel

Transplant adult male moths were tested in a sustained-flight wind tunnel between the 2^nd^ and 4^th^ hours of scotophase (16:8 L:D photoperiod). Conditions within the wind tunnel were as follows: 22°C, 60% RH, a wind speed of 0.5 m/second, and illumination of 11 lux of red light at the tunnel floor [41]. The odor source was located 1.5m from the release platform. Moths were scored for taking flight from the release cage, locking on to the pheromone plume and initiation of upwind flight (UP), continued orientation and upwind flight half way to the odor source (MID), and source contact (SC). Males were recaptured after each flight so that they could be flown to each of the 4 blends detailed above (a minimum of 30mins elapsed between each subsequent test). The order that blends were tested was randomized each day. Following flight tunnel testing each male was placed into an individual container with a small wick of cotton wool soaked in water. Males were then shipped overnight to the University of Utah.

### Chemicals (neurophysiology)

Synthetic pheromone-related compounds Z11-16:Ald, Z9-16:Ald, Z11-16:OAc, Z11-16:OH, and Z9-14:Ald were obtained from Sigma-Aldrich (St. Louis, MO) and Bedoukian Research (Danbury, CT), purities > 95% verified by gas chromatography). Serial dilutions of individual compounds were made with HPLC-grade hexane and stored at -20°C. For individual odorant stimulation cartridges, 10μl of equivalent diluted solution was loaded onto a 0.7 X 3.5 cm filter-paper strip for a total loading of 100ng. The following pheromone mixtures were also included with Z11-16:Ald loaded at 100ng and additional components in the stated ratios:

1. Z11-16:Ald, Z9-14:Ald, Z9-16:Ald, Z11-16:OH, Z11-16:OAc (1:1:1:1:1) (5-mix)
2. Z11-16:Ald, Z9-14:Ald (1:0.05) (*H*. *virescens* 2-mix)
3. Z11-16:Ald, Z9-16:Ald, Z11-16:OH, Z11-16:OAc (1:0.5:0.1:0.1 (*H*. *subflexa* 4-mix)

### Recording and Staining of Olfactory Projection Neurons

Intracellular recordings were attempted on all transplants that at least demonstrated taking flight (TF) behavior to any of the odor sources tested in the wind tunnel. The procedures of recording and staining were as previously described [23]. Once a stable penetration was established, physiological identity was tested with a series of odor stimulations (single odorants and mixtures) to the ipsilateral antenna. Each stimulus was delivered as a series of five consecutive 60ms pulses (3Hz) of humidified air (0.8 liter/min flow rate). Neuronal activity was recorded on a VHS tape PCM recorder (A.R. Vetter, Rebersburg, PA) and subsequently digitized and analyzed with DataPac 2000 software (Run Technologies, Laguna Niguel, CA).

We used three neuronal tracers to stain physiologically characterized PNs. Microelectrode tips were filled with either 3% Lucifer Yellow CH (Sigma-Aldrich, St. Louis, MO) in a 0.2M LiCl solution backfilled with 2M LiCl, 5% Neurobiotin^™^ (Vector Lab, Burlingame, CA) in 0.25M KCl backfilled with 3M KCl, or 3% dextran tetramethylrhodamine (Molecular Probes, Portland OR) in 0.1M Phosphate Buffer solution (PB) backfilled with 0.2M PB. Tracers were injected intracellularly with negative current (~ -3nA) for Lucifer Yellow or positive current (~ 5nA) for Neurobiotin and TMR-dextran (~2nA). After fixation in 3% formaldehyde solution, brains that were stained with Neurobiotin [44] were incubated in a blocking solution (1% bovine serum albumin, 0.5% Triton X-100 in 0.2 M phosphate buffer solution) overnight, and then in 1:80 Streptavidin-Alexa Fluor 555 (Invitrogen, Grand Island, NY) diluted in blocking solution for 12hrs at 4°C and for 6hrs at RT. After rinsing, brains were dehydrated in an ethanol series and cleared with methyl salicylate. Images of 1 or 2μm thickness optical sections were collected under a Zeiss LSM 510 laser scanning confocal microscope through a Plan-Neofluor 20X objective lens (NA 0.5) after excitation with 458 nm Argon laser (LP 475 nm filter) for Lucifer Yellow, 543 nm HeNe laser excitation (554~619 nm band filter) for Alexa 555, and 543 HeNe laser excitation (560–600 nm band filter) for TMR-dextran. Post-hoc image adjustment was made with LSM Image Browser 4.2 (Carl Zeiss, Jena, Germany).

### Recording and Staining of Olfactory Receptor Neurons

The activity of ORNs in V-S transplant males was recorded using the cut-tip single sensillum recording technique [45,46] as described previously [27,47]. The tip of a 1ml disposable pipette was removed and an individual transplant male was inserted such that the head and antenna protruded through the open end. The head was immobilized in an appropriate orientation with dental wax and an Ag/AgCl ground wire was inserted into the abdomen. Randomly selected sensilla were cut using custom-made tool [47,48] constructed with a tip-broken glass recording electrode and 27mm diameter piezoelectric disc connected to a function generator. After cutting the sensilla, the custom tool was retracted and the recording electrode was advanced toward the antenna to make a connection with a single cut sensillum. A continuous stream of humidified air (1.0 liter/min flow rate) was directed over the antenna. Odorant stimuli (two consecutive 100ms pulses with 1.5s interpulse interval) were injected into the moving airstream and over the antenna in order to characterize the responses of ORNs within the target sensillum. Each stimulation cartridge contained a filter paper strip impregnated with 100μg of a pheromone-related compound (Z11-16:Ald, Z9-14:Ald, Z9-16:Ald, Z11-16:OH, Z11-16:OAc). Stimulation control, signal digitalization and signal recordings were managed with a custom-written program in Labview 6.1^®^ software (National Instruments, Austin, TX) [46].

Neuronal staining was attempted on a physiologically characterized sensillum using the cobalt-lysine staining technique [10,27,29,47,49–51]. The recording electrode was replaced with a staining electrode containing 0.5 M cobalt lysine solution and maneuvered to make contact with the tip of the target sensillum for 15 ~ 20 min. The animal was then kept in a humidified environment for 2 days at 4°C. The brain was exposed by removing the head cuticle and membranous tissues and irrigated in physiological saline (150mM NaCl, 3mM KCl, 3mM CaCl_2_, 10mM TES, and pH 7.0). After transferring into a vial with 1ml saline, the head was treated with 2 ~ 3 drops of fresh ammonium sulfide solution for 10min on the rotor, and then washed four times for 5min each with saline containing 2% sucrose. After overnight fixation in FAA fixative (formaldehyde-alcohol-acetic acid), the brain was rinsed in 95% ethanol and rehydrated for silver intensification [52]. Brains were then preliminarily counterstained using modified Lee’s Methylene Blue-Basic Fuchsin solution [27,53] for 10min to give better chromatic contrast between cell bodies and glomerular neuropil. Our modified staining solution consisted of 0.5% methylene blue + 0.5% azure II in 1% borate: 0.7% basic fuchsin in 95% ethanol: 100% ethanol = 1:2:1, or 0.15% methylene blue, 0.15% azure II, and 0.35% basic fuchsin in 73% alcohol. The stained brains were washed with 70% ethanol, and then gradually dehydrated with 95%, 100% ethanol (x2), and propylene oxide (x2). They were then infiltrated with a mixture of Durcupan resin (Sigma-Aldrich, St. Louis, MO) and propylene oxide by allowing the propylene oxide to evaporate at room temperature overnight. After transferring into pure resin, brains were oriented for frontal sections and cured at 60°C for at least 2 days. Cured brain blocks were sectioned at 10μm thickness and images digitally captured.
